# Association between preoperative diagnosis of sarcopenia and postoperative pneumonia in resectable esophageal squamous cell carcinoma patients: a retrospective cohort study

**DOI:** 10.3389/fonc.2023.1144516

**Published:** 2023-05-18

**Authors:** Zhiyun Xu, Qianwei Wang, Zhenzhong Zhang, Yaning Zhu, Yunyun Chen, Derong Tang, Jianqiang Zhao

**Affiliations:** ^1^Department of Thoracic Surgery, The Affiliated Huaian No. 1 People’s Hospital of Nanjing Medical University, Huaian, China; ^2^Department of Pathology, The Affiliated Huaian No. 1 People’s Hospital of Nanjing Medical University, Huaian, China

**Keywords:** esophageal squamous cell carcinoma, postoperative pneumonia, sarcopenia, McKewon procedure, complications

## Abstract

**Background:**

Postoperative outcomes for patients suffering from resectable esophageal squamous cell carcinoma (ESCC) are related to sarcopenia. In patients with resectable ESCC, this study investigated the link between sarcopenia and postoperative pneumonia.

**Methods:**

The McKewon procedure was the only one used to treat resectable ESCC patients from January 2018 to December 2021 in this retrospective analysis. Sarcopenia was assessed using skeletal muscles at L3 and planning CT scans. It was defined when PMI was below 6.36 cm^2^/m^2^ and 3.92 cm^2^/m^2^ for men and women, separately. Analyses of multivariate and univariate logistic regression were applied for identifying the risk factors for postoperative pneumonia.

**Results:**

The study included 773 patients with resectable ESCC in total. Sarcopenia was an independent risk factor for postoperative pneumonia in individuals with resectable ESCC based on univariate and multivariate analysis (P < 0.05). The stratified analysis indicated that neither of the clinical outcomes in the logistic regression model were affected by gender, age, BMI, smoking, or pre-albumin (P for interaction > 0.006).

**Conclusion:**

Following the McKewon procedure, patients with resectable ESCC who were sarcopenic had a higher postoperative pneumonia rate. To prevent the development of postoperative pneumonia during the perioperative period, it may be important to control the incidence of sarcopenia.

## 1 Introduction

Due to its complicated nature, poor prognosis, and high mortality, esophageal cancer places a significant social burden on society (1, 2). Incidence of esophageal cancer in China ranks sixth among malignant tumors, with the fourth highest mortality rate, accounting for more than half of all cases (3, 4). Esophageal squamous cell carcinoma (ESCC) is the major subtype of esophageal cancer, representing 95% of the diagnosed esophageal cancer (5). Treatment options for ESCC mainly include radiotherapy or chemotherapy or esophagectomy, with the goal of curative resection (6, 7). Surgery is still the primary treatment, especially for patients suffering from early-stage esophageal cancer (8, 9). The traditional esophagectomy is among the most invasive surgical procedure, and the current mainstream treatment types of minimally invasive esophagectomy (MIE) include McKeown procedure (cervical anastomosis) as well as Ivor Lewis procedure (intrathoracic anastomosis) with the advent of thoracoscopic/laparoscopic esophagectomy (TLE) (10, 11).

Although reported rates are close to 60% (12, 13), the rate of postoperative complications linked to MIE continues to be significant. Postoperative pneumonia is among the most frequent complications after MIE, which accounts for 25-30% of all cases (14, 15). Postoperative pneumonia is frequently associated with prolonged hospitalization and a poor long-term prognosis (16, 17). According to current research, malnutrition, diabetes mellitus, advanced age, increased intraoperative blood loss, thoracotomy, obesity, together with recurrent laryngeal nerve palsy (RLNP), these are all the risk factors for postoperative pneumonia in esophageal cancer patients (18–21). Improved predictors of post-operative pneumonia in individuals receiving MIE are required as these indicators have not been widely accepted as essential predictors of the condition.

Recently, it has been identified that loss of skeletal muscle mass, also known as “sarcopenia”, is a poor prognostic factor for various conditions and diseases (22–24). In cancer patients, sarcopenia is linked to more severe treatment toxicity and poorer survival (25, 26). Additionally, sarcopenia is a prominent predictor of complications following surgery for bladder, pancreatic, and colorectal cancers and liver transplantation (27–30). Sugimura et al. have reported that sarcopenia assessed through the use of the L3 skeletal muscle mass index (SMI) may be a predictor of pulmonary complications following esophagectomy (31). Besides, the psoas major muscle index (PMI) is now among the diagnostic standards for the diagnosis of sarcopenia recommended from the Asian Working Group on Sarcopenia (AWGS) together with European Working Group on Sarcopenia in the Older People (EWGSOP) (32, 33). Nevertheless, many researches have adopted SMI as a diagnostic standard for sarcopenia to assay the correlation between postoperative complications and sarcopenia, while PMI has been rarely utilized. In contrast to SMI, PMI features the advantages of clear boundaries, small measurement area, and single muscle measurement (34). As a result, the measurement is simpler and more precise. Simultaneously, there has been little research into the ability of sarcopenia based on PMI diagnosis to predict postoperative pneumonia in patients with ESCC treated with McKewon procedure alone.

As a result, the purpose of this work is to clarify, using the McKeown approach, the predictive value of sarcopenia based on PMI diagnosis for postoperative pneumonia following MIE.

## 2 Materials and methods

### 2.1 Study design and participants

A retrospective cohort study was implemented within the Department of Thoracic Surgery, Huai’an No. 1 People’s Hospital, Nanjing Medical University, Nanjing, China, from January 2018 to December 2021. In the current work, patients had resectable esophageal squamous cell carcinoma and underwent only a thoraco-laparoscopic esophagectomy, which is a minimally invasive procedure. Patients with either of the following conditions will be excluded/included from the trial. The exclusion criteria are listed below: (A) comorbidity with other malignancies; (B) preoperative CT imaging data not available; (C) patients receiving neoadjuvant therapy; (D) perioperative clinicopathological data not available.

Inclusion criteria are as below: (A) Patients receiving digestive tract endoscopy prior to surgery and were pathologically confirmed to have squamous cell carcinoma;

Patients confirmed as squamous cell carcinoma by digestive endoscopy before surgery; (B) CT imaging data of chest and abdomen scan within one week before operation in our hospital. All patients were clinically staged in accordance with the eighth edition of the TNM classification. The study followed the Declaration of Helsinki guidelines and was authorized through the Ethics Committee of Nanjing Medical University.

### 2.2 Information on clinical parameters

Retrospectively obtained clinical parameters from medical records included demographics, co-morbidities, medication information, and laboratory data. The calculation of BMI is weight (kg) divided by height squared (m^2^). Normal weight is measured by a 18.5 kg/m^2^ BMI, it is then measured by a BMI between 18.5 and 24 kg/m^2^, and overweight (with a BMI of 24 kg/m^2^) (35). The cut-off point for stratifying prealbumin was the identification of low prealbumin by prealbumin <160 mg/L (36). Diagnostic criteria for the diagnosis of postoperative pneumonia within thirty days of surgery should conform to the following three criteria in the meantime: (A) at least two chest radiographs and at least one diagnosis of pneumonia; (B) at least meet the following one, age ≥70 years, peripheral blood WBC count <4 x 10^9^/L or >12 x 10^9^/L, and fever (the temperature of body >38°C) with altered consciousness; (C) at least two of the following, such as the emergence of purulent sputum or sputum properties change, or increased respiratory secretions, or need to increase the number of sputum, or dyspnea.

### 2.3 Determination of sarcopenia according to PMI index

Prior to surgery, abdominal computed tomography (CT) scans from ESCC patients were applied for calculating the skeletal muscle mass. The software Philips Vue PACS (Philips Electronics UK LTD, Farnborough, UK) was used to retrospectively analyze CT images in our institution. The muscles were measured in the HU range between -29 and +150 HU, and any necessary manual corrections to the tissue boundaries were made. PMI was estimated after summing the right and left psoas muscle areas in the L3 segment and normalizing it to the height of the patient. (Bilateral psoas area/height^2^). In accordance with the new diagnostic standard recommended for low skeletal muscle mass on CT imaging in Asian adults, we applied the gender-specific PMI thresholds for sarcopenia of 3.92 cm^2^/m^2^ and 6.36 cm^2^/m^2^ for women and men, separately (37).

### 2.4 Statistical analysis

Baseline pathological and clinical variables were expressed as range and median for consecutive variables and as proportion and frequency for categorical variables. Pathological and clinical variables were examined *via* employing chi-square tests. The relation between postoperative pneumonia and sarcopenia was examined after the use of models of univariate and multivariate logistic regression analysis. Three models were employed: model 1, adjusted for age and sex; model 2, adjusted for hypertension, sex, age, smoking together with diabetes; and model 3, adjusted for hypertension, sex, age, smoking, diabetes grade, tumor location, N-stage, as well as T-stage. For identifying interactions and modifications, likelihood ratio tests and stratified logistic regression models were applied in subgroups of hypertension, age, gender, smoking, BMI, diabetes, grade, tumor location, prealbumin, N stage and T stage. All of the statistical analyses were implemented *via* applying the Free Statistics software version 1.7.1 along with the software package R (http://www.R-project.org, The R Foundation). When *P* < 0.05, the statistical differences were deemed significant.

## 3 Results

### 3.1 Baseline characteristics

**Table 1** presents the pathological and clinical characteristics of 773 participants enrolled in the work. Population-specific characteristics by postoperative pneumonia are presented in **Table 2**. 521 males and 212 females made up this cohort; 138 of the males had postoperative pneumonia. Of the females, 45 also had postoperative pneumonia. 505 participants were over the age of 65, while 228 participants were under that age. The overall cohort contained 523 patients with sarcopenia in accordance with the suggested sarcopenia diagnostic criteria, 145 of whom had pneumonia postoperatively. Postoperative pneumonia was used as a grouping variable, and BMI, smoking and sarcopenia were significantly different between groups. In comparison to the postoperative and non-postoperative pneumonia groups, the sarcopenia had more positive results (79.2% vs. 68.7%) in the postoperative pneumonia group.

**Table 1:** Clinic and pathological features of patients.

Characteristics	Number (%)
Age (year) median (range)	67 (41-88)
BMI (kg/m^2^) median (range)	22.53 (15.27-33.75)
PMI (cm^2^/m^2^) median (range)	
Male	4.915 (1.293-9.069)
Female	3.466 (1.399-4.711)
Pre-albumin (mg/L) median (range)	196.8 (33.2-353.9)
Hypertension	
Yes	196(26.7)
No	537(73.3)
Diabetes mellitus	
Yes	63(8.6)
No	670(91.4)
Smoking	
Yes	222(30.3)
No	511(69.7)
Grade	
I	160(21.8)
II	463(63.2)
III	110(15)
T stage	
T1	177(24.1)
T2	166(22.6)
T3	390(53.2)
N stage	
N0	474(64.7)
N1	156(21.3)
N2	79(10.8)
N3	24(3.3)
Tumor location	
Upper	92(12.6)
Middle	485(66.2)
Low	156(21.3)

**Table 2:** Baseline characteristics of the study population by postoperative pneumonia.

Characteristics	Total (n = 733)	Postoperative pneumonia		*P* value
		No (n = 550)	Yes (n = 183)	
Gender, n (%)				0.136
Male	521 (71.1)	383 (69.6)	138 (75.4)	
Female	212 (28.9)	167 (30.4)	45 (24.6)	
Age, n (%)				0.1
<65 years	228 (31.1)	180 (32.7)	48 (26.2)	
≥65 years	505 (68.9)	370 (67.3)	135 (73.8)	
Hypertension, n (%)				0.329
No	537 (73.3)	408 (74.2)	129 (70.5)	
Yes	196 (26.7)	142 (25.8)	54 (29.5)	
Diabetes mellitus, n (%)				0.599
No	670 (91.4)	501 (91.1)	169 (92.3)	
Yes	63 (8.6)	49 (8.9)	14 (7.7)	
BMI, n (%)				< 0.001
Underweight	48 (6.5)	27 (4.9)	21 (11.5)	
Normal weight	428 (58.4)	314 (57.1)	114 (62.3)	
Over weight	257 (35.1)	209 (38)	48 (26.2)	
Smoking, n (%)				0.049
No	511 (69.7)	394 (71.6)	117 (63.9)	
Yes	222 (30.3)	156 (28.4)	66 (36.1)	
Sarcopenia, n (%)				0.006
No	210 (28.6)	172 (31.3)	38 (20.8)	
Yes	523 (71.4)	378 (68.7)	145 (79.2)	
Pre-albumin, n (%)				0.095
≥ 160 mg/L	639 (87.2)	486 (88.4)	153 (83.6)	
< 160 mg/L	94 (12.8)	64 (11.6)	30 (16.4)	
Grade, n (%)				0.779
I	160 (21.8)	119 (21.6)	41 (22.4)	
II	463 (63.2)	351 (63.8)	112 (61.2)	
III	110 (15.0)	80 (14.5)	30 (16.4)	
T stage, n (%)				0.909
T_1_	177 (24.1)	135 (24.5)	42 (23)	
T_2_	166 (22.6)	124 (22.5)	42 (23)	
T_3_	390 (53.2)	291 (52.9)	99 (54.1)	
N stage, n (%)				0.671
N_0_	474 (64.7)	362 (65.8)	112 (61.2)	
N_1_	156 (21.3)	115 (20.9)	41 (22.4)	
N_2_	79 (10.8)	56 (10.2)	23 (12.6)	
N_3_	24 (3.3)	17 (3.1)	7 (3.8)	
Tumor location, n (%)				0.831
Upper	92 (12.6)	67 (12.2)	25 (13.7)	
Middle	485 (66.2)	364 (66.2)	121 (66.1)	
Low	156 (21.3)	119 (21.6)	37 (20.2)	

### 3.2 Univariate and multivariate analyses of postoperative pneumonia

Among all clinicopathological factors analyzed, smoking, both sarcopenia and BMI were remarkably related to postoperative pneumonia (**Table 3**). After adjustment for various confounding factors, sarcopenia has a positive correlation with the postoperative pneumonia in three models. In three models, odds ratios (ORs) for sarcopenia were persistently significant. Of note, the adjusted OR for postoperative pneumonia was 1.53 (95% CI: 1.01-2.33) (**Table 4**), when sarcopenia was assessed as the categorical variable in the all-variable adjusted model (model 3).

**Table 3:** Results of univariate logistic analysis and predictors of postoperative pneumonia.

Clinical variables	Postoperative pneumonia	
	OR.95%CI	*P* value
Gender		
Male	Ref	
Female	0.75 (0.51~1.1)	0.136
Age		
<65 years	Ref	
≥65 years	1.37 (0.94~1.99)	0.101
Hypertension		
No	Ref	
Yes	1.2 (0.83~1.74)	0.329
Diabetes.mellitus		
No	Ref	
Yes	0.85 (0.46~1.57)	0.599
Smoking		
No	Ref	
Yes	1.42 (1~2.03)	0.047
BMI		
Underweight	Ref	
Normal weight	0.47 (0.25~0.86)	0.014
Over weight	0.3 (0.15~0.57)	<0.001
Sarcopenia		
No	Ref	
Yes	1.74 (1.16~2.59)	0.007
Pre.albumin		
≥ 160 mg/L	Ref	
< 160 mg/L	1.49 (0.93~2.38)	0.097
Grade		
I	Ref	
II	0.93 (0.61~1.4)	0.716
III	1.09 (0.63~1.89)	0.763
T.stage		
T_1_	Ref	
T_2_	1.09 (0.67~1.78)	0.735
T_3_	1.09 (0.72~1.66)	0.673
N.stage		
N_0_	Ref	
N_1_	1.15 (0.76~1.74)	0.503
N_2_	1.33 (0.78~2.25)	0.294
N_3_	1.33 (0.54~3.29)	0.536
Tumor.location		
Upper	Ref	
Middle	0.89 (0.54~1.47)	0.653
Low	0.83 (0.46~1.5)	0.544

**Table 4:** Multivariable-adjust ORs and 95%CI of the sarcopenia associated with postoperative pneumonia.

Sarcopenia	Crude Model		Model 1		Model 2		Model 3	
	OR.95%CI	*P* value	OR.95%CI	*P* value	OR.95%CI	*P* value	OR.95%CI	*P* value
No	Ref		Ref		Ref		Ref	
Yes	1.74 (1.16~2.59)	0.007	1.52 (1.01~2.29)	0.047	1.51 (1~2.29)	0.048	1.53 (1.01~2.33)	0.044

Model 1 adjust for gender and age. Model 2 adjust for Model 1 and hypertension and diabetes mellitus and smoking. Model 3 adjust for Model 2 and pre-albumin and grade and T stage and N stage and tumor location.

### 3.3 Subgroup analyses by adjusted potential effect confounders

**Figure 1:**
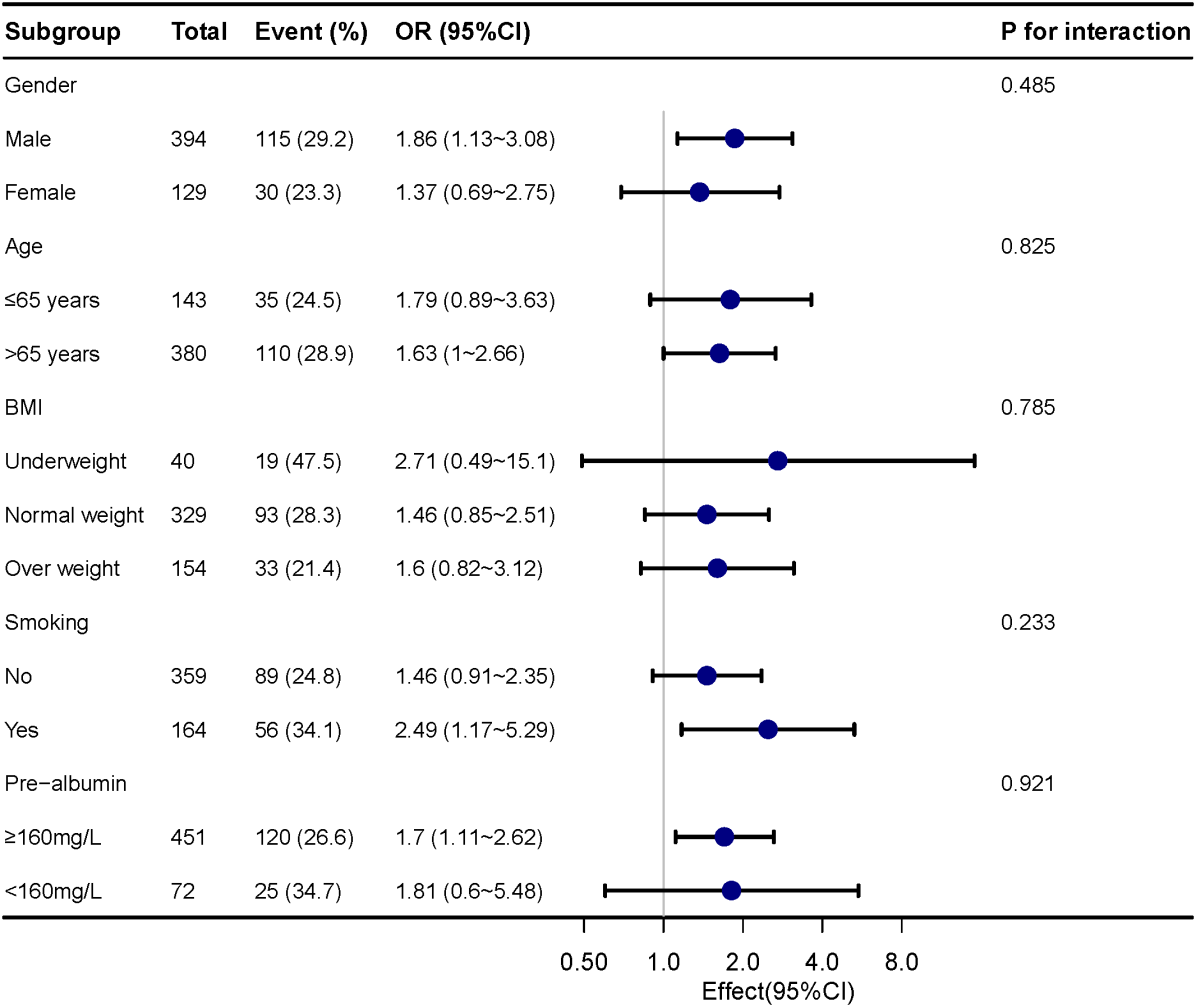
Subgroup analyses of the sarcopenia and postoperative pneumonia, stratified by gender, age, BMI, smoking and pre-albumin.

For assessing the impact of sarcopenia on pneumonia in various subgroups, subgroup analyses were carried out. The correlation between postoperative pneumonia and sarcopenia was coordinated in subgroups as below: age (≤65 years versus >65 years; P = 0.852 for interaction), gender (male versus female; P = 0.485 for interaction), prealbumin (≥160 mg/L versus <160 mg/L; P=0.921 for the interaction) and BMI (underweight versus normal weight/overweight; P = 0.785 for interaction), as shown in **Figure 1**.

## 4 Discussion

Regarding postoperative pneumonia, this work assessed the value of the prognosis of sarcopenia in ESCC patients undergoing minimally invasive McKeown esophagectomy. In the current work, we found that postoperative pneumonia in ESCC patients had an independent risk factor for sarcopenia. Additionally, sarcopenia had a consistent predictive value for postoperative pneumonia across gender, age, BMI, smoking, and pre-albumin subgroups.

To our knowledge, this is the first research to examine the correlation between the prevalence of postoperative pneumonia and sarcopenia in ESCC patients only undergoing MIE-McKeown surgery. As we all know, surgery for ESCC in complex and entails many challenges. As science and technology advance, minimally invasive surgery has gradually become the standard surgical approach globally (38). However, patients with ESCC still experience up to 38% of postoperative pulmonary complications from surgery (39). The primary causes of postoperative pneumonia in patients suffering from ESCC after esophagectomy are thought to be malnutrition and surgical stress (40, 41).

Recently, studies have started to evaluate the correlation between sarcopenia and postoperative complications in various tumors. For example, Tolga Olmez et al. suggested that sarcopenia may lead to longer Length of intensive care unit stay in colon cancer (42). Sarcopenia may cause worse overall survival in renal cell carcinoma (43). Additionally, for some malignant tumors with a long-term prognosis, sarcopenia may be a predictor of OS or DFS (44–46). The number of patients suffering from sarcopenia is growing because of the disease-specificity of patients with upper gastrointestinal cancers, which are difficult to administer orally (47). In correspondence, over 70% of the patients in our work satisfied the criteria for sarcopenia. Several prior researches depicted sarcopenia diagnosed according to L3-SMI can lead to specific adverse events in ESCC patients after surgery (48). Therefore, the increased incidence of postoperative pneumonia and expectoration disorder among patients were probably due to deterioration of strength in the respiratory and swallowing muscles (49, 50). In addition to the SMI standard, the sarcopenia diagnosed by PMI based on CT scanning has gradually gained attention in recent years. SMI was calculated from the entire skeletal muscle area in cross-section of the third lumbar vertebral body, while PMI was measured from the sum of the right and left lumbar muscle areas next to the third lumbar vertebral body, with better accuracy and lower systematic error (37). Nevertheless, few researches have examined the association between PMI-based sarcopenia as the diagnostic criteria and postoperative pneumonia in ESCC patients. In our study, sarcopenia has been identified as a risk factor for post-operative pneumonia, simultaneously remained stable in the subgroup analysis results.

After surgery for gastrointestinal cancer, weight loss and skeletal muscle wasting are inevitable, especially in the first 10 days (51). If the patient has been diagnosed with preoperative sarcopenia before surgery and have to accept surgical treatment during this period, it will often lead to many postoperative complications (52). From the point of view of postoperative pneumonia, it is essential to ameliorate sarcopenia prior to esophagectomy. Moreover, over time, interventions of exercise therapy and nutritional support prior to surgery have become possible (53). Sarcopenia-related postoperative pneumonia is thought to have a secondary systemic inflammatory response in the muscles brought on by hypoactivity, malnutrition, and surgical stress (54). The underlying mechanisms of sarcopenia-induced postoperative pneumonia is still unclear. However, the current pathogenesis of sarcopenia involves the following aspects: Decline in exercise capacity associated with age is a primary factor in the loss of muscle strength along with mass in older adults (55). Another important reason is the deterioration of neuro-muscular function. Normal function of the motor neurons is critical to the viability of muscle fibers (56). Moreover, the alpha motor neuron loss is a critical factor in sarcopenia pathogenesis. It was discovered that the motor neurons were markedly reduced in older adults after the age of 70, while the loss of alpha motor neurons amounted to 50% (57). Furthermore, some related studies have suggested that insulin, estrogen, androgen, growth hormone and glucocorticoid changes were also involved in the pathogenesis of sarcopenia (58, 59). For example, fat in the body and muscle cells increases, which is associated with insulin resistance in sarcopenia (60). Currently, experiments have confirmed that protein anabolism is significantly reduced after aging muscle cells receive insulin (61). It was reported that pro-inflammatory cytokines were also implicated in the pathogenesis of elderly sarcopenia. The CRP, TNF-α and IL-6 levels were found to be linked to muscle strength and mass (62). In the last several years, it has been suggested that immunohistochemical markers CD34 and CD10 may be able to act as markers in basal cells, though this remains to be confirmed by further histopathology development (63–65). Muscle biopsy shows that muscle cell apoptosis in the elderly is significantly higher than in young people, which indicated that myocyte apoptosis may be related to mitochondrial dysfunction and muscle mass loss (66). Studies have confirmed that type II muscle fibers mainly involved in sarcopenia are more likely to die through apoptotic pathways (67). And, it was also reported that aging, oxidative stress, low growth factors, and complete immobilization can induce caspase-dependent or -independent apoptotic signaling pathways (68).

This study presents several limitations. First, it was a retrospective cohort study that was implemented at a single institution, which might have introduced a potential selection bias. Second, all cases in this research were all squamous cell carcinomas. Therefore, the enrolled population studied has potential selection bias. The strength of this work is that we have the maximum sample size for the analysis of the association of postoperative pneumonia in ESCC patients currently studied, with only McKinsey surgery treatment.

In summary, our findings indicate that sarcopenia is markedly related to postoperative pneumonia in resectable ESCC patients. More interest should be given to the perioperative nutritional status in patients with a preoperative diagnosis of “sarcopenia”. Further researches are required to verify these results.

## Data availability statement

The original contributions presented in the study are included in the article/supplementary material. Further inquiries can be directed to the corresponding authors.

## Ethics statement

The study was performed the Declaration of Helsinki guidelines and was approved by the Ethics Committee of Nanjing Medical University. The patients/participants provided their written informed consent to participate in this study.

## Author contributions

JZ, DT, and ZX designed the study. QW and ZZ collected the data. YZ and YC analyzed the data. ZX, QW, DT, and JZ interpreted the result. ZX wrote the first draft of the manuscript. ZZ contributed to the refinement of the manuscript. All authors contributed to the article and approved the submitted version.
